# Testing and Calibration of CDs as Radon Detectors at Highly Variable Radon Concentrations and Temperatures

**DOI:** 10.3390/ijerph16173038

**Published:** 2019-08-22

**Authors:** Dobromir Pressyanov, Luis Santiago Quindos Poncela, Strahil Georgiev, Ivelina Dimitrova, Krasimir Mitev, Carlos Sainz, Ismael Fuente, Daniel Rabago

**Affiliations:** 1 Faculty of Physics, “St. Kliment Ohridski”, Sofia University, 1164 Sofia, Bulgaria; 2 Radon Group, University of Cantabria, 39005 Santander, Cantabria, Spain

**Keywords:** radon, CD-method, blind comparison, extremely variable concentrations, unstable temperature

## Abstract

The application of the compact disk (CD) method for radon measurements at mines, caves and other workplaces needs testing under highly variable exposure conditions. We present the results from a blind comparison of CDs exposed in the Laboratory of Natural Radiation (Saelices el Chico, Spain). During the exposure the temperature varied from 6.5 to 24.9 °C (average 12.6 °C) and the ^222^Rn activity concentrations varied from <10 Bq m^−3^ to 147 kBq m^−3^. Good correspondence was observed between the integrated ^222^Rn activity concentration determined by the reference instruments in the laboratory (122,500 ± 6100 kBq h m^−3^) and that assessed by analysis of the CDs at a depth 80 µm beneath the front surface (118,000 ± 12,000 kBq h m^−3^) and at a depth of 120 µm (106,000 ± 12,000 kBq h m^−3^). The theoretical modeling of the CD response under variable temperature and radon concentration suggested that the small bias is probably due to the time variation of the calibration factor because of the time variations of the temperature.

## 1. Introduction

The compact disk (CD) method for radon measurements was proposed in 2001 [1], initially as a method for retrospective measurements. It is based on radon absorption in the polycarbonate material of which CDs and digital versatile disks (DVDs) are made and analysis of alpha tracks at a certain depth beneath the disk surface (higher than 76 µm, usually about 80 µm) as described elsewhere [1,2]. Since 2001 the method has been thoroughly studied in the laboratory and in indoor radon surveys [2]. The temperature is the only identified environmental factor to have an effect on the results, and it can be corrected for a posteriori [2]. Past comparisons made indoors showed good correspondence between the CD method and conventional measurements [2]. However, new applications of this method (e.g., for measurements in mines [3] or caves) require tests of the method under more extreme conditions than those typically found indoors. The comparison of results obtained by CDs under extremely high variations in the radon activity concentration and variable temperature with parallel measurements by reference radon monitors can test the potential of the method for applications at peculiar working places or environmental conditions. Here we describe the results of a blind comparison of radon measurements by CDs and continuous radon monitors, which was carried out in the Laboratory of Natural Radiation (LNR) located in Saelices el Chico (Salamanca, Spain). This is a unique laboratory facility where radon activity concentration can vary by orders of magnitude and in which continuous follow-up of radon activity concentrations and environmental parameters (temperature, humidity, pressure) is made by reference instruments [4].

## 2. Materials and Methods

**Figure 1:**
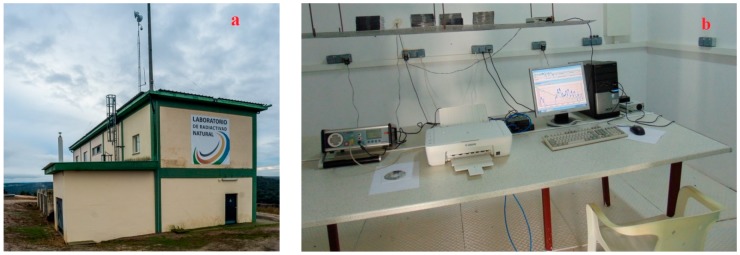
(**a**) Photos of the Laboratory of Natural Radiation (**b**) and the place in Room 1 where the experimental exposure was carried out.

The LNR was set up and handled by the University of Cantabria (Figure 1a). It is located inside the former uranium mine of Saelices el Chico (Salamanca, Spain) managed by the Spanish National Uranium Company ENUSA, currently under reclamation process. It has been used for calibration and testing of instruments and detectors for the measurement of natural radiation under environmental conditions. The ground floor has two spaces designed as radon chambers (Room 1 and Room 2) with approximately 45 m^3^ volume each. Room 1 has no direct connection to the exterior while Room 2 has an artificial ventilation system installed but switched off during the experiment. The radon source is the uranium mine underground soil which has a high radium content. 

During the blind test a set of 10 CDs (verbatim, recordable) were exposed in Room 1 (Figure 1b) for 171 days from 29 September 2017 to 19 March 2018. The disks were exposed in their “jewel cases” (the protective boxes in which CDs or DVDs are usually stored). The jewel cases are not hermetic, and radon penetrates freely inside them. It has been experimentally proved that CDs exposed to ^222^Rn bare and in their jewel cases give statistically identical results [1,5]. The radon activity concentration and some major environmental parameters were followed continuously (every 10 min) by a reference instrument AlphaGUARD PQ2000 PRO (Saphymo/Bertin Instruments, Frankfurt am Main, Gernany) traceable to another AphaGUARD unit calibrated in the Physikalisch-Technische Bundesanstalt (PTB). The reference instrument was verified at the LaRUC’s radon chamber (Laboratory of Environmental Radioactivity, University of Cantabria) [6].

**Figure 2:**
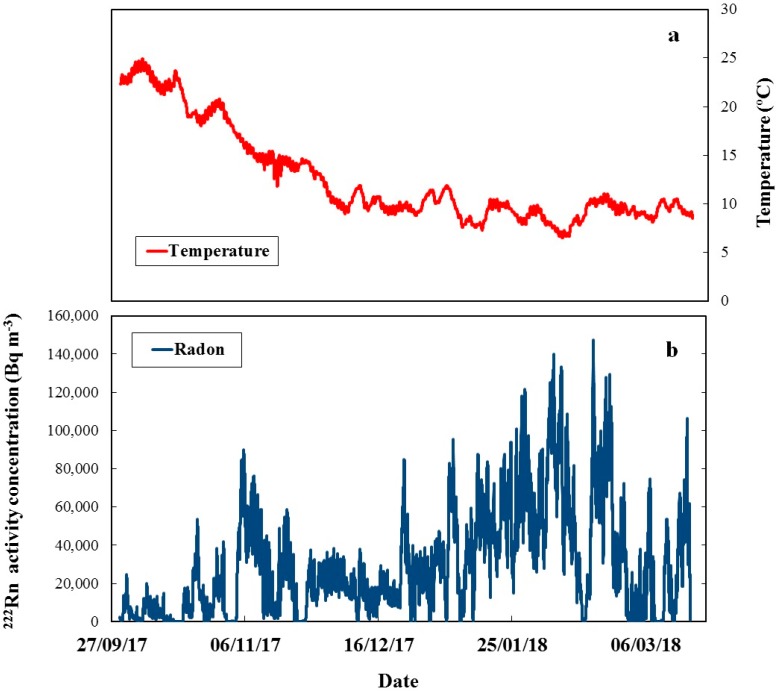
(**a**) Temperature during the exposure. The average temperature was 12.6 °C; (**b**) ^222^Rn activity concentration during the exposure. The concentration varied from <10 to 147,000 Bq m^−3^.

**Figure 3:**
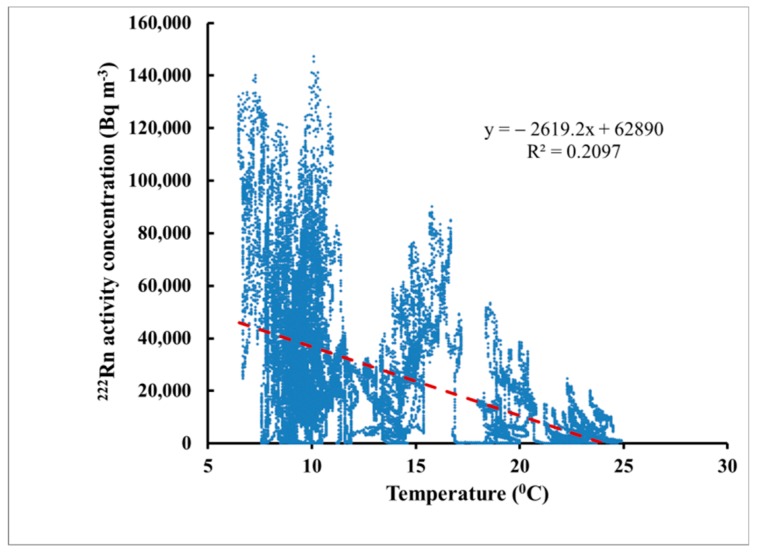
The correlation between the temperature and the ^222^Rn activity concentration. The statistical analysis made by PAST statistical package [7] showed a statistically significant (at 95% level of confidence) negative correlation.

The average temperature during the exposure was 12.6 °C (range 6.5–24.9 °C, Figure 2a), the average pressure was 944 hPa (903.6–960.2 hPa) and the average relative humidity as 64.4% (27.5%–97.4%). The radon activity concentration varied by orders of magnitude: from <10 to 147,000 Bq m^−3^ (Figure 2b). The variations in radon concentration levels were irregular, while those in the temperature showed a systematic pattern modified by irregular fluctuations. There was a weak negative correlation between the temperature and ^222^Rn activity concentration (Figure 3). However, at any temperature ^222^Rn levels can vary in a wide range, therefore the temperature variations are not considered as the primary cause for ^222^Rn variations. The ^222^Rn activity concentrations measured by the reference monitor in the LNR were exchanged with the Sofia University team once the final results were obtained (after the CDs calibration, etching and analysis). The temperature variations were shared previously as they were needed to calibrate the CDs at the mean temperature.

After exposure the disks were processed at Sofia University, Bulgaria. The processing starts with chemical pre-etching, in order to reach the desired depths (in this case 80 µm and 120 µm) by chemical removal of the surface layer. After that, electrochemical etching is applied and the tracks are counted automatically. The etching procedure is described in detail in [2] and the automatic track counting by a computer scanner in [8]. The analyzed signal is the net track density (the track density after the background is subtracted). The background of unexposed CDs of the kind used in the experiments was 3.8 ± 1.3 cm^−2^.

**Figure 4:**
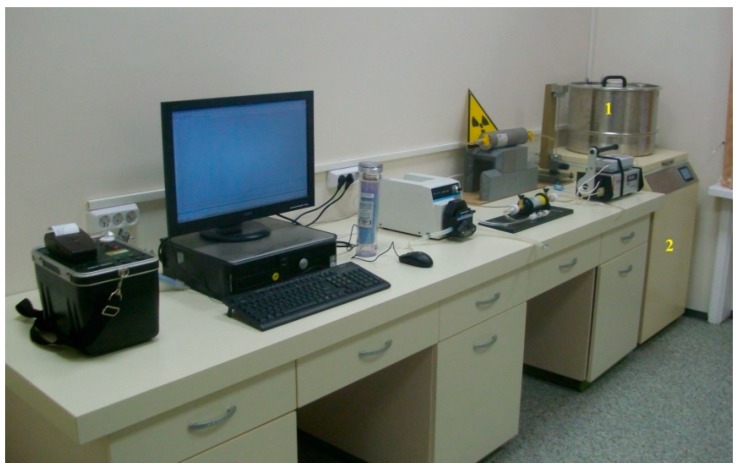
A photo of the exposure facility [9]. The detectors for calibration are placed in the 50 L exposure box (1) that is placed in the programmable thermostat (2).

The calibration of the CDs was carried out at Sofia University, Bulgaria by exposure of identical unexposed disks at reference radon concentrations at the average temperature of the exposure in the LNR (12.6 °C). The calibration exposure was done using the calibration facility described in [9] (Figure 4). The reference concentration was measured by the reference monitor AlphaGUARD PQ2000 PRO (Saphymo/Bertin Instruments, Frankfurt am Main, Germany). The calibration factor (*CF* = net track density/radon exposure) was determined for two depths beneath the disk surface: 80 µm and 120 µm. The *CF* values at the average temperature were as follows:

*CF* (80 µm) = 0.00946 ± 0.00054 cm^−2^/kBq h m^−3^

*CF* (120 µm) = 0.00286 ± 0.00024 cm^−2^/kBq h m^−3^

Since the track density decreases in depth [8], the *CF* at temperature 12.6 °C at depth 80 µm is 3.3 times greater than the *CF* at 120 µm. Analysis at depths greater than 80 µm can be useful when the signal at 80 µm is high and approaches the saturation level. While it is hard to analyze the tracks in a saturated track detector, the CDs give the opportunity to analyze them at a greater depth at which the tracks are less and to ensure quantitative measurements.

## 3. Results and Discussion

**Figure 5:**
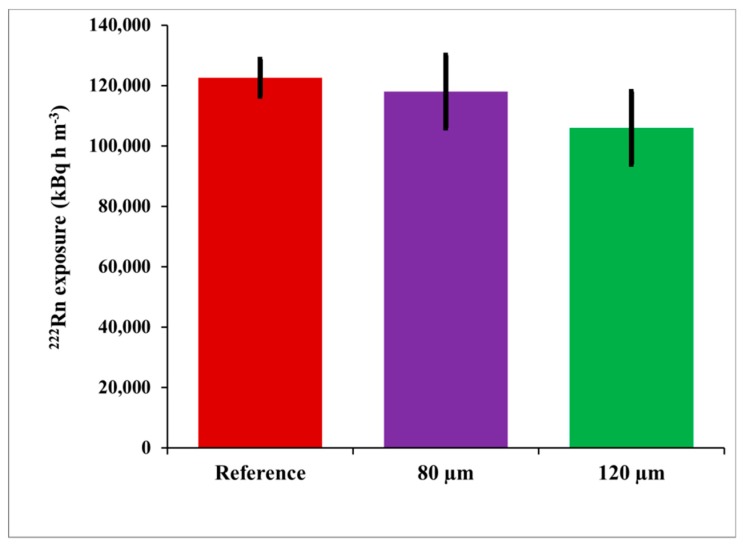
^222^Rn exposure assessed by reference measurements, compact disks (CDs) analyzed at 80 µm and 120 µm deep.

**Figure 6:**
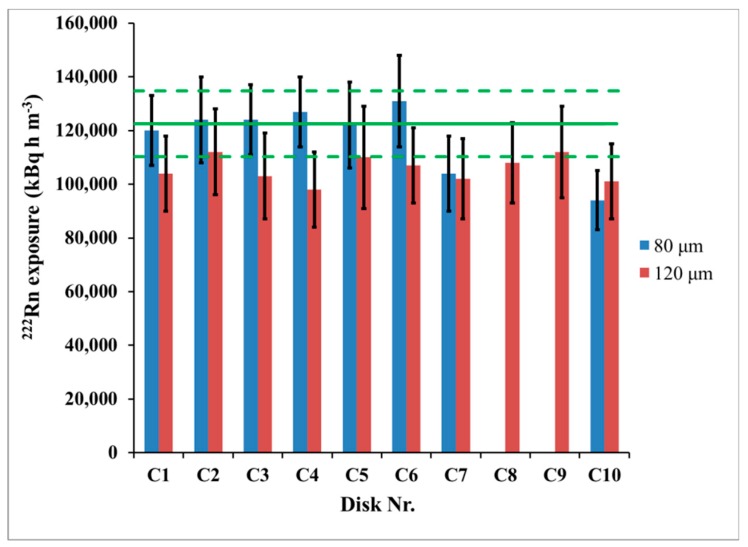
Variation of individual results between disks from one set at depths 80 µm and 120 µm. Disks C8 and C9 were analyzed only at 120 µm. The error bars correspond to the “one sigma” combined uncertainty (counting uncertainty and calibration uncertainty). The horizontal line represents the reference ^222^Rn exposure and the dashed lines show its 95% confidence interval (“two-sigma” interval).

The integrated ^222^Rn activity concentration (^222^Rn exposure) was determined by numerical integration of the values of the ^222^Rn activity concentration measured by the reference continuous monitor. Its value for this experiment was *I* = 122,500 ± 6100 kBq h m^−3^. The ^222^Rn exposure by CDs was determined by the net track-density at two depths beneath the CD surface, 80 µm and 120 µm, considering the obtained calibration factors. The results of the blind comparison are illustrated in Figure 5. The individual results for the ^222^Rn exposure by the single CDs analyzed at 80 µm and 120 µm are shown in Figure 6.

**Figure 7:**
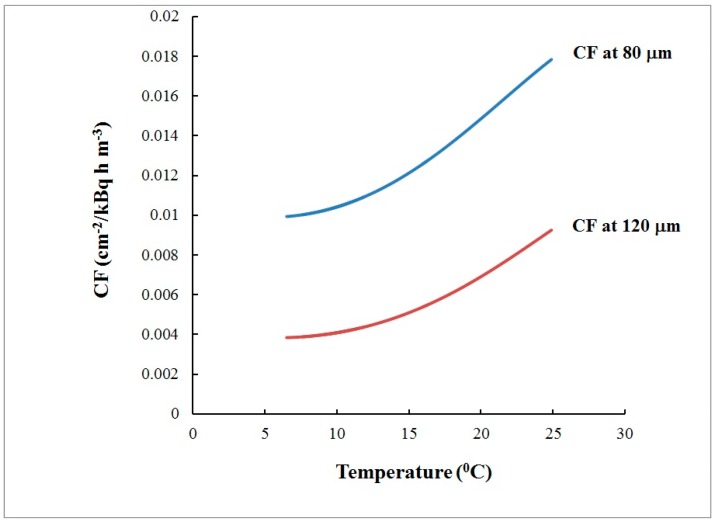
Dependence of the modeled calibration factor [11] on the temperature within 6.5–24.6 °C at depths of 80 µm and 120 µm.

The differences between the reference activity concentration and that assessed by CDs were 3.7% at 80 µm and 13.5% at 120 µm (Figure 5). The *t*-test [7,10] showed that they are not statistically significant at 95% level of confidence. However, a small and systematic bias was observed at both depths analyzed. Therefore, after the results from the blind comparison became available, we explored potential reasons for such bias. The CD calibration factor depends on the temperature, and the time variations of the temperature may incur bias in the results obtained by using the *CF* value estimated during the calibration exposure at “the average” temperature. To study this bias, theoretical modeling which follows the model described in [11] was employed. In the theoretical model [11] the dependence of the *CF* is modeled analytically and numerically as a function of the temperature within the temperature interval 5–38 °C. The model [11] considers the radon absorption and the track-etch properties of the polycarbonate material of which the commercial CDs/DVDs are made. The temperature dependence of the *CF(T)*, modeled for the studied temperature interval according to [11] is illustrated in Figure 7 for the two depths at which the signal is analyzed: 80 µm and 120 µm. 

**Figure 8:**
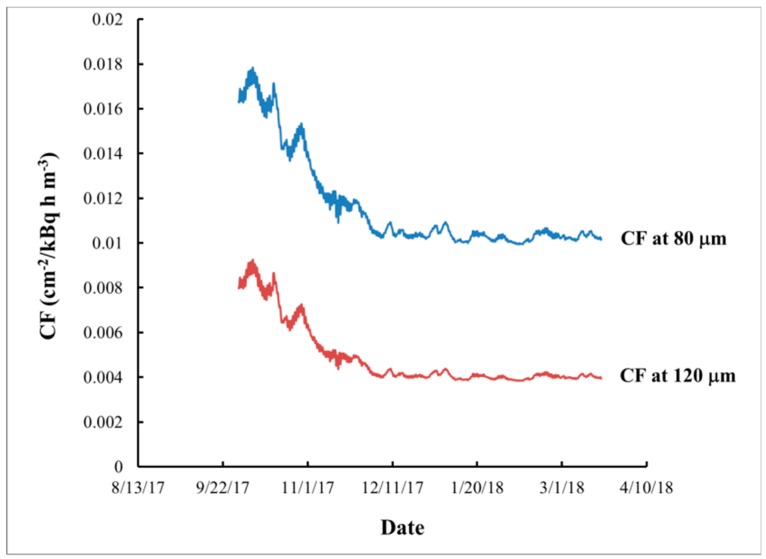
Dependence of the modeled calibration factor [11] on time at depths of 80 µm and 120 µm.

In the real exposure the calibration factor depends on the temperature *T*, which depends on the time *t*. By combining the temperature dependence of the *CF*(*T*) with the time dependence *T*(*t*) of the temperature (Figure 2a), the time dependence of the calibration factor *CF*(*T*(*t*)) can be determined (Figure 8). On the other hand, the ^222^Rn activity concentration *C_A_*(*t*) also depends on the time (Figure 2b). The “true” calibration factor $\bar{CF}$ is the ratio between the signal *n* and the ^222^Rn exposure (*I*) at the specific exposure conditions (i.e., $n=\bar{CF}I$). Any small time interval *dt* at which *CF*(*t*) and *C_A_*(*t*) can be considered practically constant contributes to the signal by $dn=CF\left(t\right)C_{A}\left(t\right)dt.$ Therefore, for the signal one obtains the following expression, used in the modeling below:

(1) $$n=\bar{CF}.I=\bar{CF}\overset{t_{exp}}{\underset{0}{\int }}C_{A}\left(t\right)dt=\overset{t_{exp}}{\underset{0}{\int }}dn=\overset{t_{exp}}{\underset{0}{\int }}CF\left(t\right)C_{A}\left(t\right)dt$$

where *t_exp_* is the exposure time. The “true” calibration factor $\bar{CF}$ depends on the exposure scenario and may differ from the calibration factor $CF\left(\bar{T}\right)$ at the average temperature $\bar{T}$, where:

(2) $$\bar{T}=\frac{1}{t_{exo}}\overset{t_{exp}}{\underset{0}{\int }}T\left(t\right)dt$$

To study the effect of the eventual difference between the “true” calibration factor and that used in the blind comparison (determined in the laboratory and corresponding to the average temperature) a model approach was used. The “true calibration factors” were calculated for the known exposure conditions, by adjusting the calibration factors at the average temperature for the real exposure profile. The obtained results are:

$$\bar{CF}\;(80\;\mu m,\;\mathrm{true}\;\mathrm{exposure}\;\mathrm{profile})\;=\;0.966\cdot CF(80\;\mu m,\;12.6\;{}^{\circ}C)$$

$$\bar{CF}\;(120\;\mu m,\;\mathrm{true}\;\mathrm{exposure}\;\mathrm{profile})\;=\;0.958\cdot CF(120\;\mu m,\;12.6\;{}^{\circ}C)$$

The results of the integrated ^222^Rn activity concentrations without and with such adjustment are shown in Table 1.

As seen, adjustment for the real exposure temperature improves the correspondence between the results, making it almost perfect for CDs etched at a depth of 80 µm (deviation reduced from 3.7% to 0.4%). For CDs etched at 120 µm, the deviation between the results and the reference value is reduced from 13.5% to 9.8%. The theoretical modeling revealed that the influence of the temperature variability is greater at a depth of 120 µm and therefore greater temperature bias can be expected. However, there are situations in which the analysis at a greater depth may be preferred. At a depth of 80 µm the “upper limit” of the method (corresponding to track density saturation) is at an integrated ^222^Rn activity concentration about 260,000 kBq h m^−3^ [5]. However, the upper limit can be increased significantly by etching at a greater depth and/or by modifying the etching regime [12]. This adds the possibility to make the upper limit of this method quite greater than that of the conventional radon detectors. Thereby, the method is applicable for the measurement of very large radon exposures, either for a long exposition time or at very high radon activity concentration.

According to the results from the experimental comparison and theoretical modeling, a possible reason for the bias between the reference value and the CD results in the blind comparison could be the great time variations of the temperature and ^222^Rn activity concentration. However, this bias appears to be small even under these extreme variations. 

## 4. Conclusions

In this work, a blind test of the CD method for radon measurement under extreme conditions is presented. There is a very good correspondence between the results obtained by CDs and the reference value despite the large variations in the activity concentration of radon and the temperature and the high integrated radon activity concentration. The observed small systematic bias of 3.7% at 80 µm and 13.5% at 120 µm is explained by the significant variability of the temperature and ^222^Rn concentrations during exposure. In conclusion, when an appropriate temperature correction is applied, the CD method provides a reliable estimate of the integrated radon concentration even under extremely variable conditions. This might be important for the public health at least in two directions: (1) The CD method is usable for retrospective measurements, which are directly related to the radon risk as it is due to the exposure received in the past; (2) since there is a new legislation requiring measurements of radon in workplaces, one can find situations with very high ^222^Rn levels at which the standard detectors become saturated. By using this new technique, we minimize the probability for this, because the upper limit of the CD method is substantially higher than that of the widely used commercial detectors. Further investigations will focus on the effect of variable temperature at different exposure scenarios.

## Figures and Tables

**Table 1:** Integrated ^222^Rn activity concentration assessed by CDs with tracks analyzed at 80 µm and 120 µm beneath the front surface. The reference exposure was assessed by continuous measurements by a reference instrument AlphaGUARD PQ2000 Pro. *CF* = net track density/radon exposure.

Scenario	^222^Rn Exposure (kBq h m^−3^)		
	At 80 µm	At 120 µm	Reference
With *CF* at 12.6 °C	118,000 ± 12,000	106,000 ± 12,000	122,500 ± 6100
With *CF* adjusted for the real exposure	122,000 ± 12,000	110,500 ± 12,000	
